# Development of high-utilization honeycomb-like α-Ni(OH)_2_ for asymmetric supercapacitors with excellent capacitance†

**DOI:** 10.1039/c8ra08019d

**Published:** 2018-11-05

**Authors:** Shaojie Zhou, Shizhong Cui, Wutao Wei, Weihua Chen, Liwei Mi

**Affiliations:** Center for Advanced Materials Research, Zhongyuan University of Technology Zhengzhou 450007 P. R. China mlwzzu@163.com; College of Chemistry and Molecular Engineering, Zhengzhou University Zhengzhou 450001 P. R. China chenweih@zzu.edu.cn

## Abstract

The low utilization rate of active materials has been a critical obstacle for the industrialization of ultracapacitors. In this study, a thin layer of cross-structured ultrathin α-Ni(OH)_2_ nanosheets was successfully grown *in situ* on the surface of a nickel foam as a high-conductivity framework by a vibratory water bath route under a low temperature (80 °C) and mild conditions. Combining the ultrathin α-Ni(OH)_2_ nanosheets and ultrashort electron transport, the strategy of a perfect intercalation structure of α-Ni(OH)_2_ and a thin layer of active material on a continuous conductive framework resulted in a high utilization rate of active material, which further achieved high specific capacitance of 213.55 F g^−1^ at 1 A g^−1^ in a two-electrode system and high capacitance retention from three to two electrode system (753.79 F g^−1^ at 1 A g^−1^ in the three-electrode system). Meanwhile, the device also achieved high energy density of 74.94 W h kg^−1^ at power density of 197.4 W kg^−1^ and still retained 24.87 W h kg^−1^ at power density of 3642 W kg^−1^.

## Abstract 2

The low utilization rate of active materials has been a critical obstacle for the industrialization of ultracapacitors.

## Graphical abstract

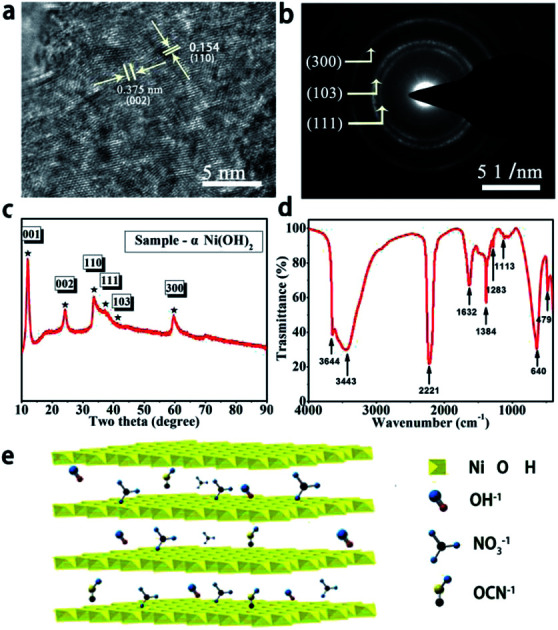

## 1 Introduction

The sustainable application of clean energy is a feasible solution to energy crisis, subject to the development of energy storage technology.^1,2^ Compared with batteries, supercapacitors (SCs) are promising storage devices due to their desirable properties such as rapid charge–discharge, excellent cycle performance, high power density and environmental safety.^3–6^ In this context, electrode materials have attracted scientific attention for use in SCs. In recent years, the strategies of micro/nano-architecture and composites with highly conductive materials were used to effectively improve the utilization of electrode material and reduce the concentration and electrochemical polarization during reversible electrochemical reactions.^7^ Nevertheless, a rapid redox reaction still occurs only on the surface of electrode materials.^8,9^ Therefore, it is very meaningful to improve the depth of the electrochemical reaction and the utilization rate of electrode materials.

Among the SC materials, α-Ni(OH)_2_ has been regarded as a scalable alternative material due to its layer structure with adjustable layer spacing depending on intercalated anions, which endows α-Ni(OH)_2_ with ideal ionic conductivity.^10,11^ Thus, OH^−^ can be transferred rapidly within α-Ni(OH)_2_. Furthermore, α-Ni(OH)_2_ can be reversibly converted into the γ-NiOOH phase without any mechanical deformation, which is helpful to improve the structural stability of electrode materials.^12^ Unfortunately, α-Ni(OH)_2_ is unstable and readily transforms to β-Ni(OH)_2_.^12,13^ Therefore, it is very difficult to prepare α-Ni(OH)_2_. For example, α-Ni(OH)_2_ nanobristles with good electrochemical stability were synthesized by diaphragm-assisted synthesis, and hierarchical α-Ni(OH)_2_ containing ultrathin nanosheets was prepared *via* solvothermal process under 120 °C.^14^ These reports provide feasible methods for the preparation of α-Ni(OH)_2_. However, special equipment and high-temperature conditions are not conducive to large-scale synthesis. Thus, there still remains a huge challenge to prepare α-Ni(OH)_2_ through mild and low-temperature methods.

Another challenge to improve the depth of the electrochemical reaction is to increase the ratio of active nickel ions that the electrons can transmit to.^15^ Based on previous reports, the combination of α-Ni(OH)_2_ and a highly conductive material is considered one of the more effective methods to improve the ratio of active nickel ions because of the increase in contact area between active material and the conductive material.^16–18^ The specific capacitances of carbon nanotube/α-Ni(OH)_2_ composites are 5.2 times that of α-Ni(OH)_2_ material at 20 mV s^−1^. However, the specific capacitance of a carbon nanotube/α-Ni(OH)_2_ composites//reduced graphene oxide device is only 78 F g^−1^ at 2 A g^−1^.^19^ This may be due to the discontinuity of the conducting network constructed by carbon nanotubes and the thickness of the active material exceeding the effective reaction depth, which result in limited ability to increase the ratio of active nickel ions. Therefore, a novel electrode material constructed by a thin layer of α-Ni(OH)_2_ growing on the surface of a continuous high-conductivity framework *in situ* can have a high utilization rate of the active material.

Herein, we report the synthesis of ultrathin α-Ni(OH)_2_ nanosheets grown on a Ni foam through a vibratory water bath route under low-temperature and mild conditions. The vibratory growing environments maintain the ultrathin nanosheets uniformly and ensure tight contact with the Ni foam, leading to the continuity of the electron transfer pathway. Meanwhile, the nanosheets show a cross-linked structure with a thickness of only 15 nm and offer numerous active surface atoms together with ultrashort electron transport to the material. A dramatic intensification of both active nickel ions and electrical conductivity demonstrates the high utilization rate for active material, which further exhibits excellent electrochemical performance with 213 F g^−1^ at 1 A g^−1^ in the two-electrode system. The promising performance of ultrathin α-Ni(OH)_2_ materials is expected to be achievable in large-scale systems.

## 2 Experimental

### 2.1 Materials and reagents

Ni(NO_3_)_2_·6H_2_O and urea were supplied by Sinopharm Chemical Reagent Co., Ltd. The compounds in this experiment were directly used without any further purification. The Ni foam was cut into a 1.5 cm × 1.5 cm cube and then washed using acetone, ethanol, and deionized (DI) water under ultrasonication for 20 min.

### 2.2 Preparation process of Ni(OH)_2_/NF

In this paper, Ni(OH)_2_ nanosheets were uniformly grown on a Ni foam by a shaking bath method. First, 0.4362 g of Ni(NO_3_)_2_·6H_2_O and 1 g urea were added to a beaker with 20 mL of deionized water. After stirring for 20 min, the above solution was transferred to a test tube with a Ni foam and heated at 80 °C in a shaking bath with a rotating speed of 200 rpm for 1 h. Finally, the Ni Foam was washed several times with deionized water and ethanol and dried at 60 °C overnight in an oven.

### 2.3 Preparation of activated carbon (AC) negative electrode

First, 90 wt% of AC and 10 wt% of PVDF were mixed in ethanol and isopropanol with a ratio of 1 : 1 and then, the solution was mixed under ultrasonication for 30 min. Next, the above solution was dropwise added and compressed into a Ni foam current collector (1.5 cm × 1.5 cm). After drying in a vacuum oven at 60 °C, we could obtain the mass loading of AC of about 30 mg.

### 2.4 Material characterization

X-ray diffraction (XRD) patterns were recorded on a Bruker D8 Advance X-ray powder diffractometer with Cu-Kα irradiation at a scan rate of 0.1° s^−1^ with 2*θ* ranging from 10° to 90°. The nanostructures of the products were recorded with a JEOL JEM-2010 transmission electron microscope (TEM). Morphologies of the sample were characterized using a Zeiss Merlin Compact scanning electron microscope (SEM) equipped with an energy dispersive X-ray spectroscopy (EDX) system.

### 2.5 Electrochemical measurements

The cyclic voltammetry (CV) and electrochemical impedance spectroscopy (EIS) tests were performed by using the electrochemical workstation (CHI660E, Chenhua, Shanghai, China). The Ni(OH)_2_/NF electrode as the working electrode, a saturated Hg/HgO electrode as the reference electrode and a platinum electrode as the counter electrode were assembled for three-electrode measurements. Galvanostatic charge/discharge (GCD) measurements were tested by using the LAND battery test system (CT2001A), and detection of two-electrode and three-electrode was operated in 2 M KOH solution.

## 3 Results and discussion

**Fig. 1:**
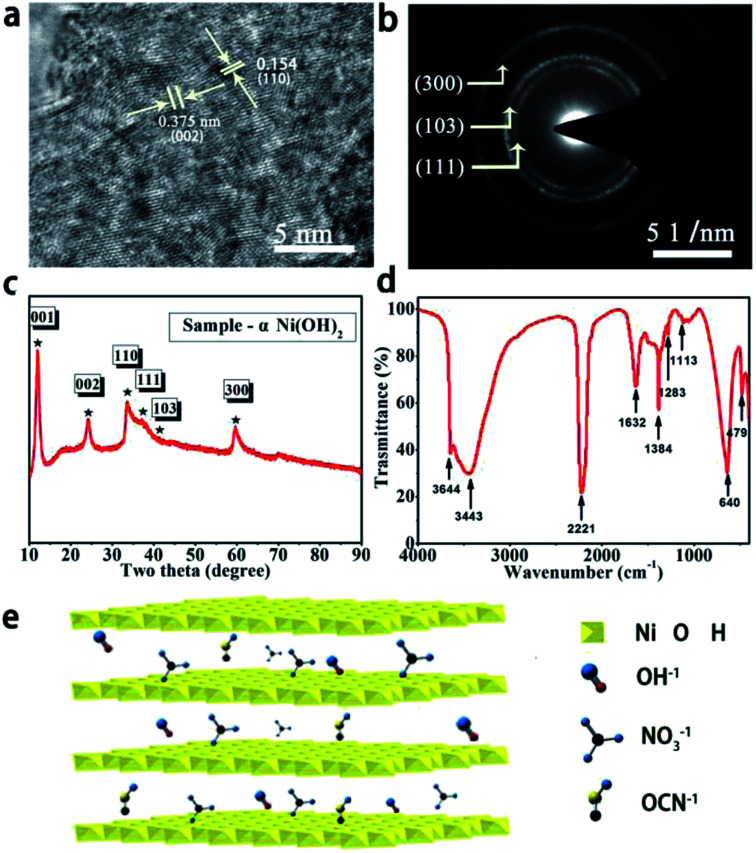
Structural characterization of α-Ni(OH)_2_ nanosheets. (a) HRTEM images, (b) SAED pattern, (c) XRD pattern, (d) FTIR spectrum, (e) and crystal structure of α-Ni(OH)_2_ nanosheets.

HRTEM was carried out to investigate the crystal structure of α-Ni(OH)_2_. The results displayed distinct lattice fringes of around 0.154 nm and 0.375 nm corresponding to the (110) and (002) planes of α-Ni(OH)_2_, as shown in Fig. 1a. The electron diffraction (SAED) patterns on selected areas in Fig. 1b show bright rings, and they were indexed to lattice planes of (111), (103) and (300), indicating high crystallinity of α-Ni(OH)_2_. XRD was further employed for confirming the phase and structure of α-Ni(OH)_2_, as shown in Fig. 1c. Several peaks were located at 11.79°, 24.32°, 33.76°, 35.24°, 41.14° and 59.74°, corresponding to (001), (002), (110), (111), (103) and (300), and these results were similar to previously reported observations.^16^Fig. 1d shows the FTIR spectrum of α-Ni(OH)_2_. The peaks at 3644.17 cm^−1^ and 640.2 cm^−1^ corresponded to ν-OH stretching and δ-OH vibrations. The peaks located at 3443.77 cm^−1^ and 1632.71 cm^−1^ were mainly assigned to the vibration of H_2_O present in the intercalation space of α-Ni(OH)_2_. The peak at 479.61 cm^−1^ was associated with the Ni–OH stretching vibration for α-Ni(OH)_2_. Clear absorption peaks at 2221.16 cm^−1^ and 1384.4 cm^−1^ indicated the presence of OCN^−^ and NO_3_^−^ anions, respectively.^20–22^ The above results illustrated that even α-Ni(OH)_2_ nanosheets with a crystalline structure and OCN^−^ and NO_3_^−^ anion intercalation can be successfully produced *via* a water bath oscillation method under low temperature and mild conditions. Fig. 1e shows a diagram of the crystal structure of α-Ni(OH)_2_ nanosheets. There are many anions in the interlayer of α-Ni(OH)_2_, which can explain the phenomenon observed in the FTIR spectrum.

**Fig. 2:**
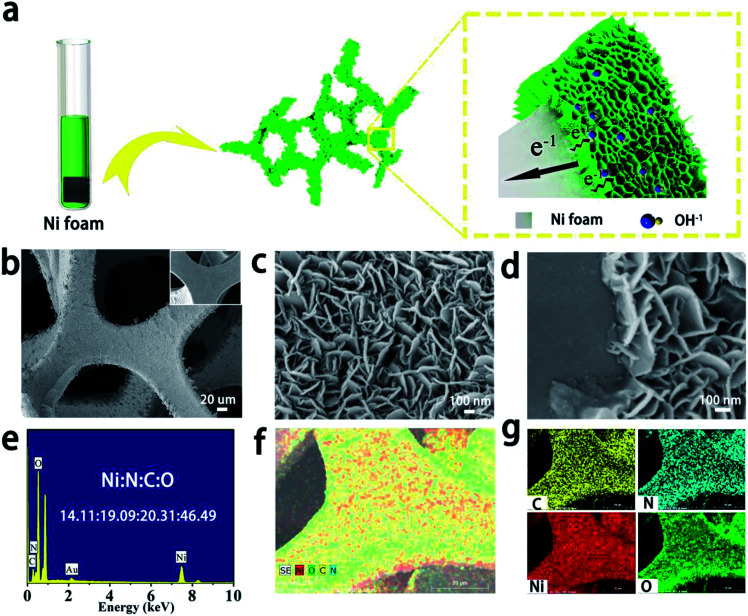
Synthesis, SEM, EDS and mapping of α-Ni(OH)_2_ nanosheets. (a) Sketch map of preparation of α-Ni(OH)_2_ nanosheets. (b–d) SEM images. (e) Elemental contents of Ni, N, C and O. (f and g) Distribution of Ni, N, C and O.

The SEM results of the samples are shown in Fig. 2. A schematic illustration of the preparation process of the sample is shown in Fig. 2a. Ultrathin α-Ni(OH)_2_ nanosheets with cross-linked structure are grown *in situ* on a Ni foam. The thin layer of α-Ni(OH)_2_ enhances the exposure of active nickel ions, which can now feasibly contact with the KOH electrolyte. Then, the electrons can be easily transferred between nanosheets and the current collector. The inset in Fig. 2b exhibits the surface of Ni foam after purification, which is smooth and clean. Compared to the SEM image of Ni foam after α-Ni(OH)_2_ formation, we can observe that a 3D skeleton architecture of Ni foam is successfully coated with α-Ni(OH)_2_ (Fig. 2b).

To further observe the morphology of α-Ni(OH)_2_ nanosheets, a higher magnification SEM image was obtained (Fig. 2c). The surface of Ni foam is uniformly covered by interlaced nanosheets with a thickness of around 15 nm. It can also be observed that there is a relatively wide space between each nanosheet, which is beneficial for electroactive materials. However, the SEM image of pure α-Ni(OH)_2_ powder exhibits a high degree of aggregation (Fig. S1†), which vastly decreases active sites and electrical conductivity. Fig. 2d shows thin nanosheets with thickness of around 120 nm on the surface of the substrate, and α-Ni(OH)_2_ nanosheets have close contact with Ni foam, which is conducive for electron transfer.

To analyse the elemental distributions of the products, EDS mappings of the as-obtained material were measured (Fig. 2e and f). Fig. 2e shows strong diffraction peaks of Au, Ni, N, C and O. The mole ratio of Ni, N, C and O is ∼14.11 : 19.09 : 20.31 : 46.49. The presence of C, N and O is mainly due to the introduction of OCN^−^ and NO_3_^−^ anions in the intercalated structure of α-Ni(OH)_2_, which is consistent with the FTIR spectrum. Nevertheless, the proportion of C and N in the intercalated structure of α-Ni(OH)_2_ is higher than that reported previously.^23–25^ The mole ratio of Ni, N and C is close to 1 : 1 : 1. As a result, the mass of OCN^−^ and NO_3_^−^ anions may be about two-thirds in the sample. Thus, the mass of the authentic active material Ni(OH)_2_ is low as anions occupy most of the mass. Fig. 2f and g show the detected area of mapping for the sample, where the distribution of Ni, N, C and O is relatively uniform, which can be proved from the skeleton with even colour.

**Fig. 3:**
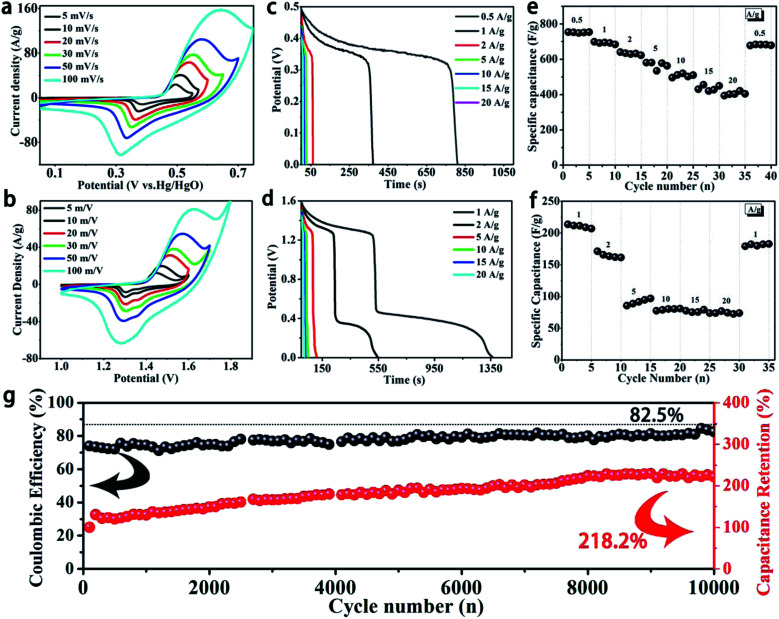
Electrochemical performances of the sample α-Ni(OH)_2_ in a three-electrode system and two-electrode system. (a and b) CV curves, (c and d) galvanostatic discharge–charge curves, (e and f) rate performance and (g) cycle testing of α-Ni(OH)_2_/NF//AC device at 5 A g^−1^.

Fig. 3a and b show CV curves of α-Ni(OH)_2_ at various scan rates from 5 to 100 mV s^−1^ in two-electrode and three-electrode systems, respectively. All curves show a pair of strong redox peaks. The appearance of the redox peaks corresponds to reversible Faradaic reactions of Ni(OH)_2_ and NiOOH; the process can be explained by the following equations:

1 Ni(OH)_2_ + OH^−^ → NiOOH + H_2_O + e^−^

2 NiOOH + H_2_O + e^−^ → Ni(OH)_2_

Furthermore, the strong current response can be due to the exposure of numerous active nickel ions between thin Ni(OH)_2_ nanosheets and the electrolyte. Meanwhile, the continuous conductive network constructed by thin nanosheets and Ni foam is beneficial to achieve fast transportation of electrons. With an increase in scan rate, the anodic peaks and cathodic peaks shift toward higher potential and lower potential at the same time, respectively. This may be due to electric polarization.

Various current densities of GCD curves of α-Ni(OH)_2_ nanosheets in three-electrode and two-electrode systems are shown in Fig. 3c and d, respectively. Good discharge platforms can be distinctly observed in all GCD curves. This is primarily due to the contribution of fast redox reactions, and it agrees well with the CV results. Two discharge platforms in discharge curves can be observed in the two-electrode system, corresponding to electrochemical behaviour of the CV curves. Fig. 3e and f show the specific capacitance of the α-Ni(OH)_2_ nanosheet electrode at different current densities. The specific capacitance of the α-Ni(OH)_2_ nanosheet electrode can be calculated by GCD curves as follows:

3 *C*_device_ = *IΔ*_td_/*m*Δ*V*

4 *C*_electrode_ = *C*_device_/4

Here, *C*_device_ and *C*_electrode_ are the specific capacitances (F g^−1^) of the device and α-Ni(OH)_2_ nanosheet electrode, respectively; *I* is the discharge current (A), *Δ*_td_ is the discharge time (s), and Δ*V* is the potential window (V).

According to the above equations, the Ni(OH)_2_ electrode in the three-electrode system shows specific capacitances of 753.79, 699.05, 640.11, 581.05, 562.21, 430.07 and 402.18 F g^−1^ at 0.5, 1, 2, 5, 10, 15 and 20 A g^−1^, respectively. The specific capacitance is well below the theoretical capacity of Ni(OH)_2_ because of low Ni active sites. However, the active sites can exhibit a high utilization. The Ni sites were effectively utilized and the capacitance reached a maximum. The results of pure α-Ni(OH)_2_ powder in a three-electrode system are similar to the above-mentioned observations, as shown in Fig. S2.† The Ni(OH)_2_ electrode in the two-electrode system shows relatively high specific capacitances of 213.55, 171.18, 88.66, 78.88, 77.30 and 76.84 F g^−1^ at 1, 2, 5, 10, 15 and 20 A g^−1^, respectively. The Ni(OH)_2_ electrode shows high specific capacitance due to close contact and fast charge transfer between thin nanosheets and the current collector. Meanwhile, the fabricated two-electrode system device without polymer binders can result in effective utilization of active materials.^16^ On the other hand, the layers of α-Ni(OH)_2_ with intercalated OCN^−^ and NO_3_^−^ anions show higher active area, which ensures sufficient contact of the electrolyte with the active substance. Here, the thin layer of α-Ni(OH)_2_ grown *in situ* on the surface and continuous high-conductivity framework further improve the utilization rate of active material, resulting in good performance. Similarly, pure α-Ni(OH)_2_ powder only shows low specific capacitances of 110.41, 86.22, 60.37, 41.00, 32.04, 30.89, 28.37 F g^−1^ at 0.5, 1, 2, 5, 10, 15 and 18 A g^−1^, respectively (Fig. S3†). This is because of low utilization in the active material.^16^ The thin nanosheets of α-Ni(OH)_2_ grown *in situ* on a continuous high-conductivity network effectively enhance the depth of the electrochemical reaction during the fast Faraday reaction process due to high utilization of active nickel ions. The α-Ni(OH)_2_/NF//AC device shows high specific capacitance compared to other previously reported Ni(OH)_2_-based electrodes (Table 1).

**Table 1:** Comparison of specific capacitance of α-Ni(OH)_2_/NF//AC in this study and those of some Ni(OH)_2_-based electrodes

Electrode material	Two-electrodes (specific capacitance@current density)	Three-electrodes (specific capacitance@current density)	Ref.
Ni(OH)_2_/CNT/NF	112.5 F g^−1^@2.5 mA cm^−2^	3300 F g^−1^@16 F cm^−2^	6
Ni(OH)_2_/NF	109.5 F g^−1^@0.2 A g^−1^	2322 F g^−1^@1 A g^−1^	11
Ni(OH)_2_/MnO_2_	134.5 F g^−1^@1.4 A g^−1^	2628 F g^−1^@3 A g^−1^	17
o-CNT/Ni(OH)_2_	78 F g^−1^@2 A g^−1^	1368 F g^−1^@20 mV s^−1^	19
Ni(OH)_2_/Ni foil	192 F g^−1^@0.9 A g^−1^	1765 F g^−1^@2 mV s^−1^	26
Ni(OH)_2_/ACMT	121 F g^−1^@1 A g^−1^	1568 F g^−1^@1 A g^−1^	27
H–TiO_2_@Ni(OH)_2_	150.6 F g^−1^@1 A g^−1^	306 mA h g^−1^@5 mV s^−1^	28
Ni(OH)_2_//AC	153 F g^−1^@5 mV s^−1^	2188 F g^−1^@1 mV s^−1^	29
MnCo_2_O_4_@Ni(OH)_2_	121 F g^−1^@1 A g^−1^	2154 F g^−1^@5 A g^−1^	30
MWCNT/amor-Ni(OH)_2_/PEDOT:PSS	179.8 F g^−1^@1 A g^−1^	3262 F g^−1^@5 mV s^−1^	31
Ni(OH_)2_/GNs/NF	80.71 F g^−1^@0.5 mA cm^−2^	2215 F g^−1^@2.3 A g^−1^	32
β-Ni(OH)_2_/NF	105.8 F g^−1^@2 mA cm^−2^	790.3 C g^−1^@5 mA cm^−2^	33
β-Ni(OH)_2_	74.4 F g^−1^@0.2 A g^−1^	1173 C g^−1^@2 mV s^−1^	34

Fig. 3g shows the coulombic efficiency and cycle life of an α-Ni(OH)_2_/NF//AC device at 5 A g^−1^. The results show that the coulombic efficiency of the device is 82.5%, indicating excellent reversible redox reaction, and the specific capacitance of the device exhibits sustainable growth before 8000 cycles. The capacitance of the device is maintained in a steady state, and the last capacitance retention ratio of α-Ni(OH)_2_/NF//AC reaches 218.2%. The above results can be ascribed to the surface of the active material being further infiltrated during the charge and discharge processes. This phenomenon can be explained by the SEM image of the sample (Fig. S5†) after 10 000 cycles, which displays a rough surface compared to the original morphology. This results in a larger area of active nickel ions to contact with the electrolyte. Meanwhile, this also indicates a stable structure for α-Ni(OH)_2_ nanosheets. Fig. S4† shows the pure α-Ni(OH)_2_ powder cycling stability, which demonstrates a capacitance retention rate of 50.37% after 10 000 cycles at 5 A g^−1^; this is in contrast to that of α-Ni(OH)_2_ nanosheets on Ni foam and demonstrates poor cycle performance for the asymmetric supercapacitor. The results indicate that thin nanosheets of α-Ni(OH)_2_ grown on Ni foam are conducive to achieve high specific capacitance.

The values of power density (*P*) and energy density (*E*) are important assessment criteria for supercapacitors. The power density (*P*) and energy density (*E*) of the device were calculated using the following equations:

5 *E* = 1/2 × *C*_electrode_ × Δ*V*^2^

6 *P* = 3600 × *E*/*Δ*_td_

Here, *E* is the energy density (W h kg^−1^) of the device, *C*_electrode_ is the specific capacitance (F g^−1^), *P* is the power density (W kg^−1^), Δ*V* is the potential window (V) and *Δ*_td_ is the discharge time (s).

**Fig. 4:**
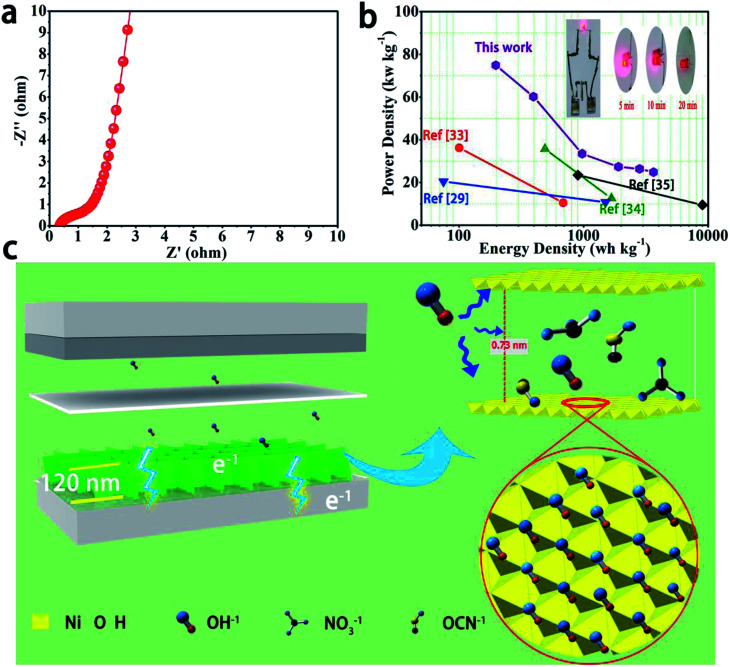
(a) Electrochemical impedance spectroscopy (EIS) of α-Ni(OH)_2_/NF//AC device. (b) The Ragone plots of the device. (c) Schematic diagram of excellent electrochemical performance of α-Ni(OH)_2_ electrode.

The Ragone plots of the α-Ni(OH)_2_/NF//AC device as asymmetric supercapacitors are shown in Fig. 4b. The device shows a high energy with 74.94 W h kg^−1^ at a power density of 197.4 W kg^−1^ and still retains 24.87 W h kg^−1^ at a power density of 3642 W kg^−1^. The results show excellent performance compared to previous literature results of β-Ni(OH)_2_/NF//AC (36.2 W h kg^−1^ at 100.6 W kg^−1^),^33^ Ni(OH)_2_/NF//AC (35.7 W h kg^−1^ at 490 W kg^−1^),^29^ β-Ni(OH)_2_//AC (20.45 W h kg^−1^ at 75 W kg^−1^),^34^ and β-Ni(OH)_2_//AC (23.45 W h kg^−1^ at 9000W kg^−1^).^35^

Practical applications of an α-Ni(OH)_2_/NF//AC device were further verified, as shown in the top right corner of Fig. 4b, where two devices were assembled in series and the LED was illuminated for up to 20 min. The schematic diagram (Fig. 4c) displays fast ion transfer and rich active sites in the electrode materials. The above results indicate the high specific capacitance of the α-Ni(OH)_2_ electrode, and the electrode can be considered as a promising candidate for power storage devices with high performance.

## 4 Conclusions

In summary, ultrathin α-Ni(OH)_2_ nanosheets were fabricated though oscillating *in situ* growth on the surface of Ni foam. Continuous high-conductive substrates were constructed between active material and Ni foam. Meanwhile, the ultrathin nanosheets with about 15 nm thickness enhanced the effective reaction depth and electron transport to the material. A high utilization rate of active material was effectively achieved, which further demonstrated the excellent electrochemical performance of this material. Therefore, the synthesis of α-Ni(OH)_2_ for energy-storage based on this strategy has great potential for supercapacitors in large-scale applications.

## Conflicts of interest

There are no conflicts to declare.

## Supplementary Material

RA-008-C8RA08019D-s001
