# Supplementary material for: Development of high-utilization honeycomb-like α-Ni(OH)2 for asymmetric supercapacitors with excellent capacitance
Source: RSC Adv. 2018 Nov 5;8(65):37129–35. doi: 10.1039/c8ra08019d (PMC9089160; doi:10.1039/c8ra08019d)
Supplement: RA-008-C8RA08019D-s001 [file RA-008-C8RA08019D-s001.pdf]

Electronic Supplementary Material (ESI) for RSC Advances.
This journal is © The Royal Society of Chemistry 2018

Development of High Utilization Honeycomb-Like α-$Ni(OH)_2$ for Asymmetric Supercapacitor with Outstanding Capacitance

Shaojie Zhou,[a] Shizhong Cui,[a] Wutao Wei,[a] Weihua Chen*[b] and Liwei Mi*[a]

[a] Center for Advanced Materials Research, Zhongyuan University of Technology, Zhengzhou, 450007 (P.R. China) ;E-mail: mlwzzu@163.com

[b] College of Chemistry and Molecular Engineering, Zhengzhou University, Zhengzhou, 450001, P. R. China. E-mail: chenweih@zzu.edu.cn

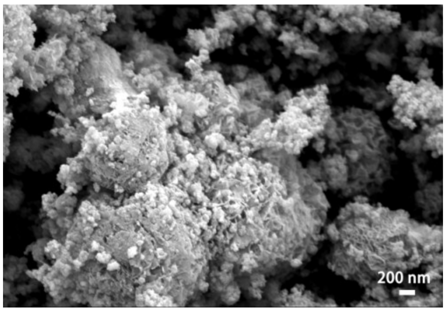

Figure S1. SEM image of pure α-$Ni(OH)_2$ power.

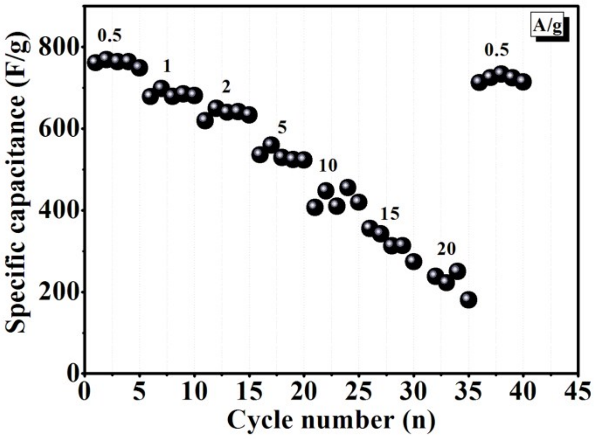

Figure S2. Rate performance of the sample pure α-$Ni(OH)_2$ power in three-electrode system.

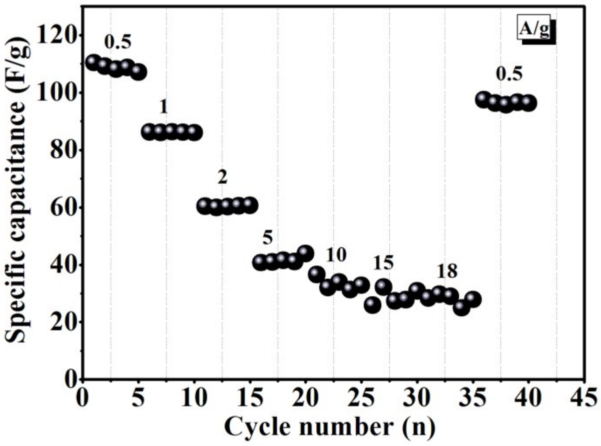

Figure S3. Rate performance of the sample pure α-$Ni(OH)_2$ power in two-electrode system.

Please do not adjust margins

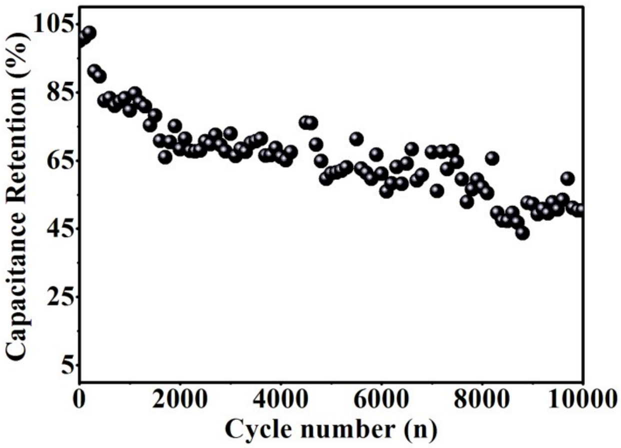

Figure S4. Cycle performance of the sample pure α-Ni$(OH)_2$ power in two-electrode system.

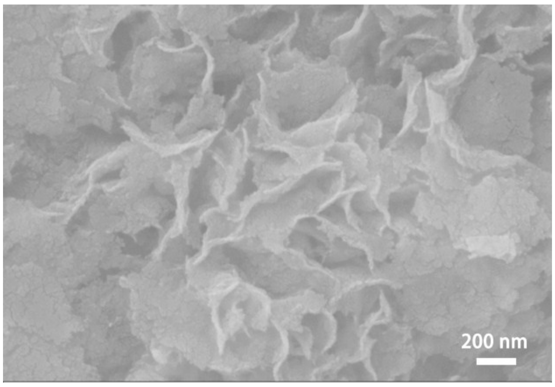

Figure S5. SEM image of the α-Ni$(OH)_2$ nanosheets of after 10000 cycle

This journal is © The Royal Society of Chemistry 20xx

Please do not adjust margins
